# ACSS2-mediated NF-κB activation promotes alkaliptosis in human pancreatic cancer cells

**DOI:** 10.1038/s41598-023-28261-4

**Published:** 2023-01-27

**Authors:** Dongwen Que, Feimei Kuang, Rui Kang, Daolin Tang, Jiao Liu

**Affiliations:** 1 The DAMP Laboratory, Third Affiliated Hospital of Guangzhou Medical University, Guangzhou, 510120 Guangdong China; 2 Department of Surgery, UT Southwestern Medical Center, Dallas, TX 75390 USA

**Keywords:** Cell biology, Cell death, Cancer, Cancer therapy, Chemotherapy

## Abstract

Alkaliptosis is a recently discovered type of pH-dependent cell death used for tumor therapy. However, its underlying molecular mechanisms and regulatory networks are largely unknown. Here, we report that the acetate-activating enzyme acetyl-CoA short-chain synthase family member 2 (ACSS2) is a positive regulator of alkaliptosis in human pancreatic ductal adenocarcinoma (PDAC) cells. Using qPCR and western blot analysis, we found that the mRNA and protein expression of ACSS2 was upregulated in human PDAC cell lines (PANC1 and MiaPaCa2) in response to the classic alkaliptosis activator JTC801. Consequently, the knockdown of ACSS2 by shRNAs inhibited JTC801-induced cell death in PDAC cells, and was accompanied by an increase in cell clone formation and a decrease in intracellular pH. Mechanically, ACSS2-mediated acetyl-coenzyme A production and subsequent histone acetylation contributed to NF-κB–dependent CA9 downregulation, and this effect was enhanced by the histone deacetylase inhibitor trichostatin A. These findings may provide new insights for understanding the metabolic basis of alkaliptosis and establish a potential strategy for PDAC treatment.

## Introduction

There are many different types of regulated cell death (RCD), which display significant morphological, biochemical, and genetic characteristics^1^. Apoptosis is the most studied form of RCD. It involves the formation of membrane-bound apoptotic bodies and is usually mediated by the caspase family. However, apoptosis resistance remains a major clinical challenge for cancer treatment^2^. Recently, several forms of non-apoptotic cell death have been reported to overcome apoptotic tolerance to treat various types of cancer^3^. Among them, alkaliptosis is a pH-dependent RCD that was first identified through drug screening to kill human pancreatic ductal adenocarcinoma (PDAC) cells^4^. The small-molecule compound JTC801 is currently a typical alkaliptosis inducer, which relies on the activation of nuclear factor kappa B (NF-κB), instead performing its well-known function as a selective antagonist of pain peptide receptors^4^. JTC801-mediated NF-κB activation inhibits the expression of carbonic anhydrase 9 (CA9), the product of which regulates pH balance and ultimately leads to alkaliptosis with plasma membrane rupture^4^. Further understanding the mechanism and regulation of pH homeostasis may improve the anticancer activity of alkaliptosis-based therapy^5^.

Acetyl-coenzyme A (AcCoA) is a metabolite that plays a central role in energy production, lipid metabolism, and epigenome modification^6^. AcCoA homeostasis directly affects the level of histone acetylation, thereby regulating gene expression^7^. AcCoA is usually produced through two pathways^6^. One is mediated by ATP citrate lyase (ACLY), which cleaves citric acid into oxaloacetate and acetyl-CoA. The other is mediated by the acetyl-CoA synthase short-chain (ACSS) family, which ligates acetate and CoA. The ACSS family includes ACSS1, ACSS2, and ACSS3, which have different subcellular locations and functions. ACSS1 and ACSS3 are mainly located in the mitochondria and participate in mitochondrial oxidative phosphorylation, while ACSS2 is mainly located in the nucleus and cytoplasm^8^. Nuclear ACSS2 recaptures the acetate released by histone deacetylation for recycling by histone acetyltransferase^9^. ACSS2 uses acetate to provide AcCoA for de novo lipogenesis and histone acetylation^10^. Although ACSS plays a role in regulating survival and apoptosis of various cancer cells^11,12^, its role in alkaliptosis is still uncertain.

In this study, we identify a new role of ACSS2 in mediating alkaliptosis in human PDAC cells by maintaining NF-κB activation and subsequently upregulating intracellular pH through histone acetylation. These findings reinforce the idea that carcinogenic or pro-survival signals can be used to trigger cell death.

## Materials and methods

### Reagents

The antibodies to ACSS2 (3658), GAPDH (5174), p-IKKα/β (2697), IKKα (11930), p-NF-κB (3033), IKBα (7217), acetylated-lysine (9814), IKKβ (8943), NF-κB (16502), p-IKBα (2859), and CA9 (5648) were obtained from Cell Signaling Technology. The antibody to PARP (13371-1-1-ap) was obtained from Proteintech. JTC801 (S272201) was purchased from Selleck Chemicals. Staurosporine (62996-74-1) and trichostatin A (58880-19-6) were purchased from MedChemExpress.

### Cell culture

PANC1 (CRL-1469), MiaPaCa2 (CRL-1420), and CFPAC-1 (CRL-1918) cell lines were obtained from the American Type Culture Collection. Cells were grown in Dulbecco's modified Eagle's medium with 10% fetal bovine serum, 2 mM of l-glutamine, and 100 U/ml of penicillin and streptomycin. The cell culture was placed in an incubator at 37 °C with 5% carbon dioxide. Cell lines were validated by short tandem repeat profiling, and routine mycoplasma testing was negative for contamination. Dimethyl sulfoxide (DMSO) was used to prepare the stock solution of drugs. The final concentration of DMSO in the drug working solution in the cells was < 0.01%. DMSO of 0.01% was used as a vehicle control in all cell culture assays^13^.

### Microarray analysis

Array hybridization was performed according to the Agilent single-color microarray-based gene expression analysis protocol (Agilent Technology)^14^. RNA quantity and quality were measured by NanoDrop ND-1000. RNA integrity was assessed by standard denaturing agarose gel electrophoresis. mRNA was purified from total RNA after rRNA removal using mRNA-ONLY eukaryotic mRNA isolation kit (Epicentre). Total RNA (100 ng) isolated from each sample was converted to cDNA, fragmented, and hybridized to GeneChip arrays. Hybridization arrays were washed, fixed, and scanned using the Agilent DNA Microarray Scanner (P/N G2505C). The GeneChip 3000 Scanner (Affymetrix) was used to scan and quantify images of hybridized GeneChip arrays. The intensity value for each probe cell in the array was calculated by the GeneChip software.

### RNAi

The pre-designed human *ACSS2*-shRNA-1 (GCTTCTGTTCTGGGTCTGAAT), *ACSS2*-shRNA-2 (CGGTTCTGCTACTTTCCCATT) and control empty shRNA (pLKO.1) were purchased from Sigma-Aldrich in a lentiviral format. We seeded 1 × 10^5^ cells in each well of a 12-well plate in 500 μl of complete medium and transduced them by lentiviral vectors at an MOI of 10:1. Transduction was carried out in the presence of polybrene (8 μg/ml). After recovering with complete culture medium, puromycin (5 µg/ml) was used for the selection of transduced cells^13^.

### Immunoprecipitation

Cells were lysed at 4 °C in ice-cold RIPA buffer (9806, Cell Signaling Technology) and cell lysates were cleared by brief centrifugation (13,000×*g*, 15 min, 4 °C). Supernatant were used for immunoprecipitation with the indicated antibodies. Generally, 2 mg antibody was added to the supernatant and incubated at 4 °C overnight. Then, 30 μl protein G or A sepharose slurry was added, and the sample was incubated for a further 2 h at 4 °C. Following incubation, beads were washed extensively with phosphate buffered saline (PBS) and proteins were eluted by boiling in 2 × sodium dodecyl sulfate sample buffer before sodium dodecyl sulfate polyacrylamide gel electrophoresis^4^.

### Western blot

Cells were lysed 1 × in cell lysis buffer (Cell Signaling Technology, 9803) with protease and phosphatase inhibitor cocktail (Cell Signaling Technology, 5872) on ice for 30 min^13^. After centrifugation at 14,000×*g* for 15 min at 4 °C, the supernatants were collected and quantified using a bicinchoninic acid (BCA) assay (Thermo Fisher Scientific, 23225). The 30 µg proteins were separated on 10% polyacrylamide gel electrophoresis (PAGE) gels (Epizyme, PG112) and transferred to polyvinylidene difluoride (PVDF) membranes (Millipore, IPVH00010). Following blocking by TBST containing 5% skim milk for 1 h, the membranes were incubated overnight at 4 °C with various primary antibodies (1:200–1:1000). After incubation with peroxidase-conjugated secondary antibodies (rabbit anti-goat IgG secondary antibody [Abcam, ab6741; 1:1000]) for 1 h at room temperature, the signals were visualized using SuperSignal West Pico PLUS Chemiluminescent Substrate (Thermo Fisher Scientific, 34580). Blots were analyzed using the ChemiDoc Touch Imaging System (Bio-Rad) and Image Lab Software (Bio-Rad). All experiments were performed at 5% CO_2_ and normoxia and were repeated at least three times, the representative results are shown.

### Cell viability and clonogenic assay

The level of cell viability was assayed using a Cell Counting Kit-8 (CCK-8) kit (YEASEN, 40203ES80) according to the manufacturer’s protocol^15^. Cells were seeded in 96-well plates (1 × 10^4^ cells/well) and treated with indicated compounds for 24 h. For each well of the plate, the medium was replaced with 100 µl of fresh medium containing 10 µl of CCK-8 solutions. The culture was then returned to the cell incubator for 60–90 min. Absorbance at 450 nm was proportional to the number of living cells in the culture. Cell viability was expressed as a relative level, and 100% cell viability was set as 1.

For the clonogenic assay, control or JTC801-treated cells were seeded in 12-well plates (500 cells per well), then the medium was changed once every 2 days. Plates were incubated at 37 °C for 3 weeks until colonies appeared. Cells were then fixed with methanol and colonies were stained with 0.4% crystal violet.

### Cell death assay

Cells were seeded at a density of 2 × 10^5^ cells/well in medium in 6-well plates. The next day, cells were incubated with the indicated treatments. After that, the cells were stained with propidium iodide (BestBio, BB-4131-1) for 20–30 min in an incubator of 5% CO_2_ at 37 °C. Morphological changes were examined using a fluorescence microscope at × 20 magnification. A Countess II FL Automated Cell Counter (Thermo Fisher Scientific) was used to assay the percentages of dead cells after propidium iodide staining. Cell death was expressed as a relative level, and 100% cell death was set as 1^15^.

### qRT-PCR analysis

Total RNA was extracted using an RNeasy Plus Micro Kit (QIAGEN, 74034) according to the manufacturer’s instructions^15^. Cell lysates were spun using the kit’s gDNA Eliminator spin columns to remove genomic DNA. Total RNA was purified using RNeasy MinElute spin columns. First-strand cDNA was then synthesized from 1 µg of RNA using PrimeScript RT Master Mix (Takara, RR036A). Then 20 µl reactions were prepared by combining 4 µl of PrimeScript RT Master reaction mix, 2 µl of gene-specific enhancer solution, 1 µl of reverse transcriptase, 1 µl of gene-specific assay pool (20 ×, 2 µM), and 12 µl of RNA diluted in RNase-, DNase-, and genomic DNA-free water. We performed qPCR using TB Green Premix Ex Taq II (Takara, RR820Q) on the C1000 Touch Thermocycler CFX96 Real-Time System (Bio-Rad). Analysis was performed using Bio-Rad CFX Manager software 2.0. The data were normalized to RNA GAPDH and the fold change was calculated via the 2^−DDCt^ method. The relative concentrations of mRNA were expressed in arbitrary units based on the untreated group, which was assigned a value of 1.

### Flow cytometeric analysis

To assess apoptosis rate in WT and ACSS2 konckdown cells after treatment with staurosporine (500 nM) or JTC801(3 µM) for 24 h, cell were collected and using Active Caspase-3/7 Staining kit (Abcam, ab284532) according to the manufacturer’s instructions. The fluorescence peak of Caspase-3/7 analyse by Flow Cytometry (BD FACSVerse, Becton,Dickinson and Company) using the FL-1channel.

For the PI staining of the cell samples, the cells were collected and resuspended in binding buffer (BD biosciences, No. 556570), and then stained with propidium iodide (2 mg/ml). The data was collected and analyzed in BD FACSVerse.

### pH assay

Extracellular pH was measured using pH meter (Seven Compact S210 pH/OPR meter, METTLER TOLEDO instrument, USA) in accordance with the manufacturer’s guidelines. Intracellular pH was determined using a fluorogenic cytoplasmic pH indicator probe (Thermo Fisher Scientific). Fluorescence intensity of the probe is then an indicator of intracellular pH. It is weakly fluorescent at neutral pH but increasingly fluorescent as the pH drops. This reagent can quantify cellular cytosolic pH in the range of 9–4 with a pKa of ~ 6.5 with excitation/emission of 509/533 nm. Subsequent use of the Intracellular pH Calibration Buffer Kit (P35379) allows this intracellular pH to be quantified. Briefly, cells were washed with Live Cell Imaging Solution (LCIS), then 10 μl of pHrodo™ Red AM or pHrodo™ Green AM was mixed with 100 μl of PowerLoad™ concentrate and was add to 10 ml of LCIS. Cells were incubated with the pHrodo™ AM/PowerLoad™/LCIS at 37 °C for 30 min and then were analyzed using the Cytation™ 5 Cell Imaging Multi-Mode Reader (BioTek, USA) using the 509/533 maxima^4^.

### Detection of acetyl-CoA

The cellular Acetyl-CoA was measured using Acetyl-CoA Assay Kit (Sigma, MAK039) according to the manufacturer’s protocol. Briefly, the cells were added with perchloric acid and homogenized. After centrifugation (10,000×*g*, 10 min, 4 °C), 400 μl supernatant was neutralized with 40 μl saturated KHCO_3_ and centrifuged at 10,000×*g* for 5 min at 4 °C. An acetyl-CoA standard curve was generated using various concentrations (0–1000 pmol/ml) of the acetyl-CoA standard provided. The background values were eliminated from the standard, and the concentration of the samples was calculated using the Cytation™ 5 Cell Imaging Multi-Mode Reader (BioTek, USA) using the 535/587 maxima.

### Statistical analysis

Data are presented as mean ± SD except where otherwise indicated. GraphPad Prism (version 8.4.3) was used to collect and analyze data. Unpaired Student’s *t* tests were used to compare the means of two groups. A one-way (for one independent variable) analysis of variance (ANOVA) with Tukey’s multiple comparisons test was used for comparison among the different groups on all pairwise combinations. The following notations were used in figures: *P < 0.05, **P < 0.01, ***P < 0.001, ****P < 0.0001; ns: not significant.

## Results

### ACSS2 is upregulated during alkaliptosis

**Figure 1:**
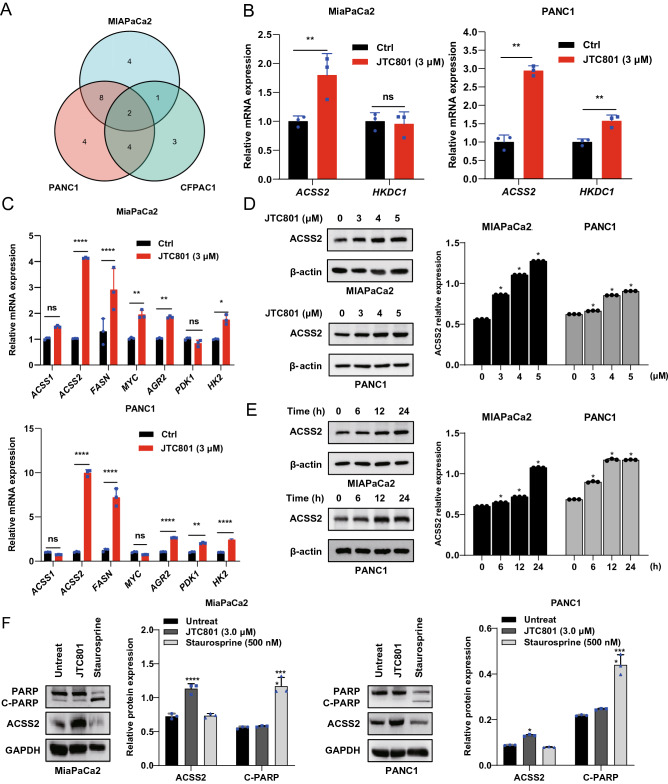
ACSS2 is upregulated during alkaliptosis. (**A**) We analyzed the upregulated genes involved in glucose metabolism and carbon metabolism in pancreatic cancer cells (MiaPaCa2, PANC1, and CFPAC-1) from DNA microarray studies after the cells had been treated with JTC801 (3 μM) for 24 h. (**B**) qPCR analysis of the mRNA of *ACSS2* and *HKDC1* in MiaPaCa2 and PANC1 cells treated with JTC801 (3 μM) for 24 h (n = 3 biologically independent samples). (**C**) qPCR analysis of the mRNA of *ACSS1*, *ACSS2*, *FASN*, *MYC*, *AGR2*, *PDK1,* and *HK2* in MiaPaCa2 and PANC1 cells treated with JTC801 (3 μM) for 24 h. (**D**) Western blot analysis of ACSS2 protein expression in MiaPaCa2 and PANC1 cells following treatment with indicated JTC801 for 24 h. The membrane were cropped and probed for indicated antibodies. Quantitative results are plotted in the right panel. (n = 3 biologically independent samples, Unpaired t-test). (**E**) Western blot analysis of ACSS2 protein expression in MiaPaCa2 and PANC1 cells following treatment with JTC801 (3 μM) for 6–24 h (n = 3 biologically independent samples, Unpaired t-test). Beta-actin was probed as a loading control. Quantitative results are plotted in the right panel. (**F**) Western blot analysis of indicated protein expression in MiaPaCa2 and PANC1 cells following treatment with JTC801 (3 μM) or staurosporine (500 nM) for 24 h. The membrane of PARP was stripped and reprobed for ACSS2. The membrane was incubated in reblot solution for 30 min and blocked for 30 min at room temperature with TBST-containing skin milk (5%) and probed overnight at 4 °C with anti-ACSS2 (Restore™ PLUS Western Blot Stripping Buffer, 46430, Thermo Scientific). Quantitative results are plotted in the right panel.

Cancer cells require a variety of metabolic reprogramming events to support their survival and rapid proliferation. These pro-survival pathways provide opportunities for designing new strategies for targeted therapies. To determine the metabolic basis of alkaliptosis, we focused on glycolysis and one-carbon metabolism, which share regulatory factors for tumor cell growth^16^. After analyzing the DNA microarray results of human PDAC (MiaPaCa2, PANC1, and CFPAC1) cells treated with JTC801, ACSS2 and hexokinase domain-containing 1 (HKDC1) were upregulated genes of glycolysis and one-carbon metabolism in all cell lines (Fig. 1A). Subsequent qPCR analysis confirmed that ACSS2 mRNA was upregulated in both MiaPaCa2 and PANC1 cells following JTC801 treatment (Fig. 1B). Since ACSS2 is mainly involved in lipid metabolism, we also used qPCR to examine key lipid metabolism genes. Other genes related to lipid metabolism, such as those of ACSS1, fatty acid synthase (FASN), MYC, pyruvate dehydrogenase kinase 1 (PDK1), and hexokinase 2 (HK2) as well as anterior gradient 2, protein disulphide isomerase family member (AGR2) were also upregulated to varying degrees in PANC1 or MiaPaCa2 cells (Fig. 1C). Moreover, western blot analysis showed that JTC801 induced the upregulation of ACSS2 protein in MiaPaCa2 and PANC1 cells in a dose- and time-dependent manner (Fig. 1D,E). In contrast, the protein level of ACSS2 was not upregulated in PANC1 cells by staurosporine (an apoptosis inducer) (Fig. 1F). Together, these findings indicate that ACSS2 is upregulated during alkaliptosis rather than apoptosis.

### ACSS2 is a positive regulator of alkaliptosis

**Figure 2:**
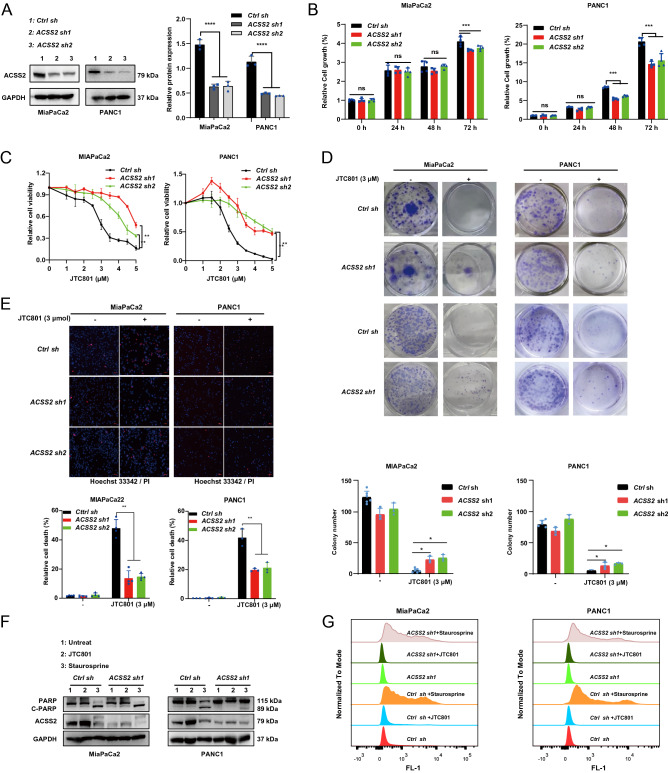
ACSS2 is a positive regulator of alkaliptosis. (**A**) Western blot analysis of ACSS2 protein expression in indicated *ACSS2* knockdown cell lines (n = 3 biologically independent samples). The membrane were cropped and probed for indicated antibodies. Quantitative results are plotted in the right panel. (**B**) Analysis of cell viability in indicated cells at 24–72 h (n = 3 biologically independent samples). (**C**) Analysis of cell viability in indicated cells following treatment with JTC801 (1–5 µM) for 24 h (n = 3 biologically independent samples). (**D**) Analysis of cell cloning formation in indicated control or JTC801-treated PANC1 and MiaPaCa2 cells. Quantitative results are plotted in the bottom panel. (**E**) Analysis of cell death by PI staining in indicated cells following treatment with JTC801 (3 µM) for 24 h (n = 3 biologically independent samples). Bar = 200 µm. Quantitative results are plotted in the below panel. (**F**) Western blot analysis of ACSS2 and C-PARP protein expression in indicated cells following treatment with staurosporine (500 nM) or JTC801 (3 µM) for 24 h (n = 3 biologically independent samples). The membrane were cropped and probed for indicated antibodies. The membrane of PARP was stripped and reprobed for ACSS2. The membrane was incubated in reblot solution for 30 min and blocked for 30 min at room temperature with TBST-containing skin milk (5%) and probed overnight at 4 °C with anti-ACSS2 (Restore™ PLUS Western Blot Stripping Buffer, 46430, Thermo Scientific). (**G**) Flow cytometeric analysis of Caspase 3/7 activity in indicated cells following treatment with staurosporine (500 nM) or JTC801 (3 µM) for 24 h (n = 3 biologically independent samples).

To determine whether the upregulated ACSS2 is implicated in alkaliptosis, we used two specific shRNAs to generate ACSS2-knockdown PDAC cells. Western blotting confirmed that in ACSS2-knockdown MiaPaCa2 and PANC1 cells, the protein level of ACSS2 was inhibited by more than 70% (Fig. 2A). Compared with the control group, the cell proliferation of ACSS2-knockdown MiaPaCa2 and PANC1 cells was inhibited at 72 h (Fig. 2B), supporting the previous finding that ACSS2 is a proliferation factor. Significantly, the knockdown of ACSS2 inhibited JTC801-induced growth inhibition in MiaPaCa2 and PANC1 cells at 24 h (Fig. 2C), highlighting a different pro-cell death role of ACSS2 in response to JTC801. Consistent with the short-term cell viability assay, the long-term cell cloning assay showed that compared with the control group, ACSS2-knockdown cells treated with JTC801 had greater proliferation ability (Fig. 2D). The use of propidium iodide (PI) staining to measure plasma membrane rupture further confirmed the hypothesis that ACSS2 is a positive regulator of alkaliptosis (Fig. 2E, Fig. S2). Consistent with previous studies^11,12^, ACSS2-knockdown cells were resistant to staurosporine-induced apoptosis as shown by a western blot analysis of cleaved poly (ADP-ribose) polymerase-1 (PARP1) and flow cytometeric analysis of Caspase-3/7 activity (Fig. 2F,G). These data establish a different role of ACSS2 in promoting JTC801-induced alkaliptosis.

### ACSS2 inhibits cytoplasm acidification in alkaliptosis

**Figure 3:**
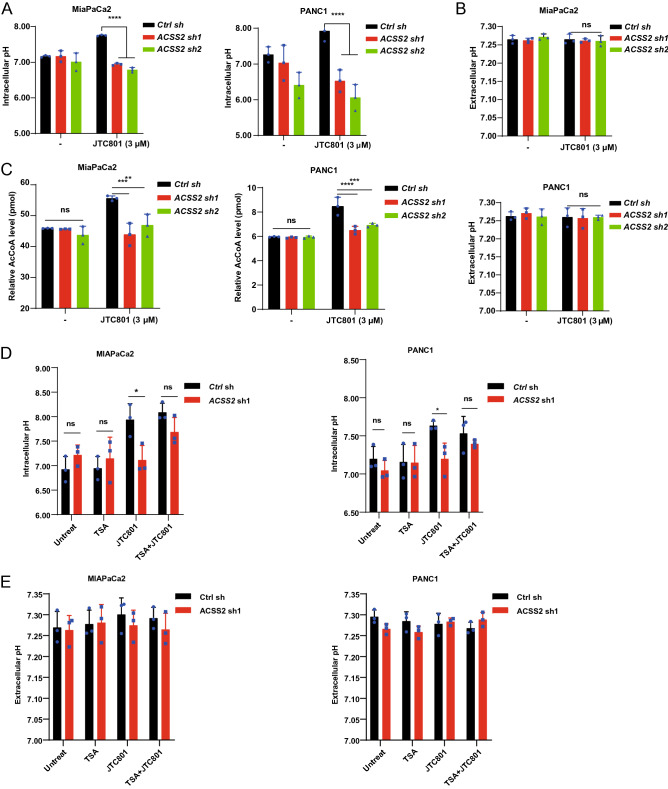
ACSS2-mediated histone acetylation inhibits cytoplasm acidification in alkaliptosis. (**A**,**B**) The intracellular and extracellular pH values were detected in indicated MiaPaCa2 and PANC1 cells after treatment with JTC801 (3 μM) for 24 h (n = 3 biologically independent samples). (**C**) ELISA analysis of acetyl-CoA levels in indicated MiaPaCa2 and PANC1 cells after treatment with JTC801 (3 μM) for 24 h (n = 3 biologically independent samples). (**D**) The intracellular pH values were detected in indicated MiaPaCa2 and PANC1 cells after treatment with JTC801 (3 μM) and trichostatin (TSA; 200 nM) for 24 h (n = 3 biologically independent samples). (**E**) The Extracellular pH values were detected in indicated MiaPaCa2 and PANC1 cells after treatment with JTC801 (3 μM) and trichostatin (TSA; 200 nM) for 24 h (n = 3 biologically independent samples).

Since increased intracellular pH is a key metabolic event that drives alkaliptosis^4^, we used a pH fluorescent probe designed to detect intracellular pH. As expected, JTC801 increased intracellular pH in MiaPaCa2 and PANC1 cells (Fig. 3A). In contrast, the suppression of ACSS2 expression significantly blocked JTC801-induced intracellular pH upregulation (Fig. 3A). As a sharp control, the extracellular pH measured by a pH test strip was not affected by JTC801 in control and ACSS2-knockdown MiaPaCa2 and PANC1 cells (Fig. 3B). These findings suggest that ACSS2 promotes alkaliptosis through the upregulation of intracellular pH rather than extracellular pH.

To further define the role of ACSS2 in alkaliptosis, we assayed the intracellular level of AcCoA. JTC801 induced the upregulation of intracellular AcCoA in control cells, but not in ACSS2-knockdown MiaPaCa2 and PANC1 cells (Fig. 3C). Trichostatin A (TSA), a broad deacetylase inhibitor that can enhance histone acetylation^17^, failed to induce intracellular or extracelluar pH upregulation in wild-type and ACSS2-knockdown cells (Fig. 3D,E). Overall, these findings indicate that ACSS2 mediates AcCoA production and intracellular pH upregulation during JTC801-induced alkaliptosis.

### ACSS2-mediated histone acetylation promotes alkaliptosis

One of the important functions of ACSS2-mediated AcCoA production is to regulate gene transcription through histone acetylation^10^. Given that NF-κB activation is an important signaling event that drives alkaliptosis^4^, we next examined the relationship between histone acetylation and the NF-κB pathway in wild-type and ACSS2-knockdown cells in response to JTC801.

**Figure 4:**
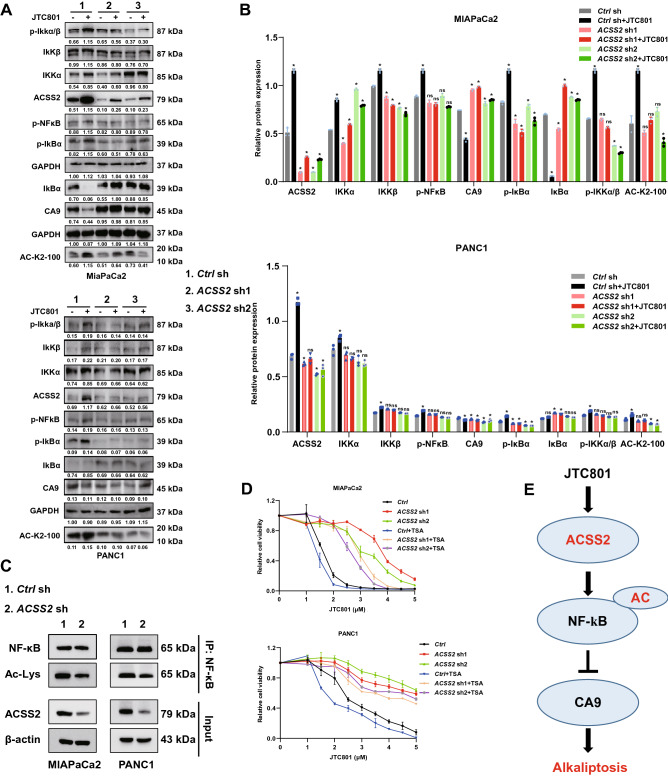
ACSS2-mediated histone acetylation promotes alkaliptosis. (**A**,**B**) Western blot analysis of indicated protein expression in control or *ACSS2* knockdown cell lines following treatment with JTC801 (3 μM) for 24 h (n = 3 biologically independent samples). Representative western blot results are shown in the left panel. The membrane were cropped and probed for indicated antibodies. The membrane of p-IKKα/β was stripped and reprobed for IKKα and IKKβ. The membrane was incubated in reblot solution for 30 min and blocked for 30 min at room temperature with TBST-containing skin milk (5%) and probed overnight at 4 °C with anti-IKKα. Repeat the above steps to reprobed the membrane with the anti-IKKβ antibody. The membrane of p-NFκB was stripped and reprobed for ACSS2. The membrane of p-IκBα was stripped and reprobed for IκBα and GAPDH. The protein expression of CA9 were measured in indicated cell lines following treatment with JTC801 (3 μM) for 24 h under 5%CO_2_ and normoxia. Quantitative results are plotted in the right panel. (**C**) Control or *ACSS2* knockdown in MIAPaCa2 and PANC1 cells. Cell lysates were extracted for coIP, the immunoprecipitates were analyzed using the indicated antibodies and representative western blot shown in the left panel. The membrane of Ac-lys was stripped and reprobed for NFκB. (**D**) Control or ACSS2 knockdown cell lines were treated with JTC801 (1–5 μM) in the absence or presence of trichostatin (TSA, 200 nM) for 24 h and then the cell viability was assayed (n = 3 biologically independent samples). (**E**) Schematic diagram of the role of ACSS2 in JTC801-induced alkaliptosis.

The IKK kinase complex is composed of two kinases (IKKα and IKKβ) and a regulatory subunit, NEMO/IKKγ, which are the core elements of the NF-κB cascade^18^. IKK phosphorylates IκBα protein, leading to subsequent IκBα ubiquitination, which is eventually degraded by the proteasome^18^. After IκBα dissociates from NF-κB (RelA and p50), activated NF-κB is transferred from the cytoplasm to the nucleus, where it starts to initiate gene transcription^18^. Our western blot results showed that JTC801 upregulates the protein expression of histone-acetylated lysine (AC-K2-100) (Fig. S3C and Fig. 3D) and the core components of the NF-κB pathway (IKKα, IKKβ, and NF-κB) in MiaPaCa2 cells (Fig. 4A,B), and cell viability assay showed that acetate increased the sensitivity of MiaPaCa2 and PANC1 cells to JTC801(Fig. S4). In contrast, the knockdown of ACSS2 reversed this process and was associated with a reduction in NF-κB phosphorylation events and an increase in CA9 expression (Fig. 4A,B). Our coIP experiments demonstrate the acetylation level of NF-κB in cell lines with knockdown of ACSS2 (Fig. 4C). We also detected the acetylation level and CA9 expression after ACSS2 knockdown by western blot (Fig. S3A and S3B). These findings indicate that ACSS2 sustains NF-κB–mediated CA9 downregulation during JTC801-induced alkaliptosis. As a positive control, CA9 was upregulated during CoCl_2_-induced hypoxia (Fig. S5).

We next examined whether TSA can reverse the inhibitory effect of alkaliptosis in ACSS2-knockdown MiaPaCa2 cells. A cell viability assay showed that TSA restored the sensitivity of ACSS2-knockdown MiaPaCa2 cells to JTC801 (Fig. 4D). In contrast, the synergy of TSA and JTC801 in wild-type cells was weak (Fig. 4D).

## Discussion

PDAC is currently the deadliest cancer of the digestive system, with an overall 5-year survival rate of about 10%. Compared with current chemotherapy that induces apoptosis, non-apoptotic RCDs (including alkaliptosis) provide new opportunities for the treatment of PDAC^19^. In this study, we demonstrated that ACSS2 is a new regulator of alkaliptosis by activating the NF-κB pathway in human PDAC cells (Fig. 4E). While ACSS2 can mediate apoptosis resistance, it is upregulated during JTC801-induced alkaliptosis in PDAC cells. These findings support that ACSS2 plays a dual role in cell death, depending on stimulus. The induction of alkaliptosis may be a valuable method for the treatment of PDAC with high ACSS2 expression.

Compared with tissue-specific ACSS1 and ACSS3, the expression of ACSS2 is widespread in different tissues^20^. Preclinical studies have shown that ACSS2 can use acetate to mediate histone acetylation, which plays a key role in regulating gene expression, cell growth, and cancer development^9,10^. In the current study, we demonstrated that JTC801-induced alkaliptosis requires this ACSS2-dependent histone acetylation pathway. Although global genetic changes of JTC801-induced alkaliptosis still need to be studied in the future, we found that NF-κB pathway expression and activation are impaired in ACSS2-knockdown PDAC cells. Like ACSS2, NF-κB is usually a pro-survival factor that inhibits apoptosis. However, alkaliptosis partly relies on the activation of NF-κB to inhibit the expression of CA9, leading to an increase in intracellular pH^4^. Increased intracellular pH may further accelerate overall histone acetylation, which is dependent on monocarboxylic acid transporters^21^. Overall, these findings establish a potential strategy for using pro-survival signals or pathways to trigger cell death in PDAC cells.

In addition to mediating the epigenetic regulation of gene transcription by histone acetylation, another important function of the ACSS family, including ACSS2, in tumor biology is to mediate fatty acid synthesis for tumor growth^6^. Fatty acid synthesis is the production of fatty acids from AcCoA and NADPH by FASN. Nevertheless, excessive fatty acid synthesis also plays a role in mediating cell death by activating lipid peroxidation-dependent ferroptosis. In particular, the polyunsaturated fatty acid biosynthesis pathway plays an essential role in ferroptosis^22^. However, early studies have shown that ferroptosis inhibitors, such as ferrostatin-1, cannot inhibit JTC801-induced cell death^4^. Therefore, although different RCDs may share some signals, their effectors and feedback mechanisms are different.

Alkaliptosis is generally a type of regulated necrosis with a rupture of the plasma membrane and release of damage-associated molecular patterns (DAMPs). High-mobility group box 1 (HMGB1) is a representative nuclear DAMP in a variety of RCDs, which can trigger various immune responses. For example, HMGB1 can be released by cancer cells in response to chemotherapy or radiotherapy to activate surrounding antigen-presenting cells to achieve antitumor immunity^23^. We recently observed that HMGB1 is a mediator of the inflammatory response caused by alkaliptotic injury^24^. In addition to passive release, active secretion of HMGB1 into the extracellular space requires the acetylation of HMGB1^25^. It is important to further examine whether the acetylation and the release of HMGB1 are dependent on ACCS2 upregulation in alkaliptosis, which may favor the development of immunotherapies for PDAC.

In summary, we demonstrated that ACCS2 is a new regulator of alkaliptosis by activating histone acetylation. These findings underscore the potential significance of ACCS2 as a biomarker and a contributor to alkaliptosis-mediated treatment^5^.

## Supplementary Information

Supplementary Information 1.

Supplementary Information 2.

Supplementary Information 3.

## Data Availability

Microarray data are deposited in GEO under accession GSE206290 https://www.ncbi.nlm.nih.gov/geo/query/acc.cgi?acc=GSE206290 Enter token ynipqmqkvrmlrux into the box.
