# Supplementary material for: ACSS2-mediated NF-κB activation promotes alkaliptosis in human pancreatic cancer cells
Source: Sci Rep. 2023 Jan 27;13:1483. doi: 10.1038/s41598-023-28261-4 (PMC9883393; doi:10.1038/s41598-023-28261-4)

Fig 1. Western blot raw data

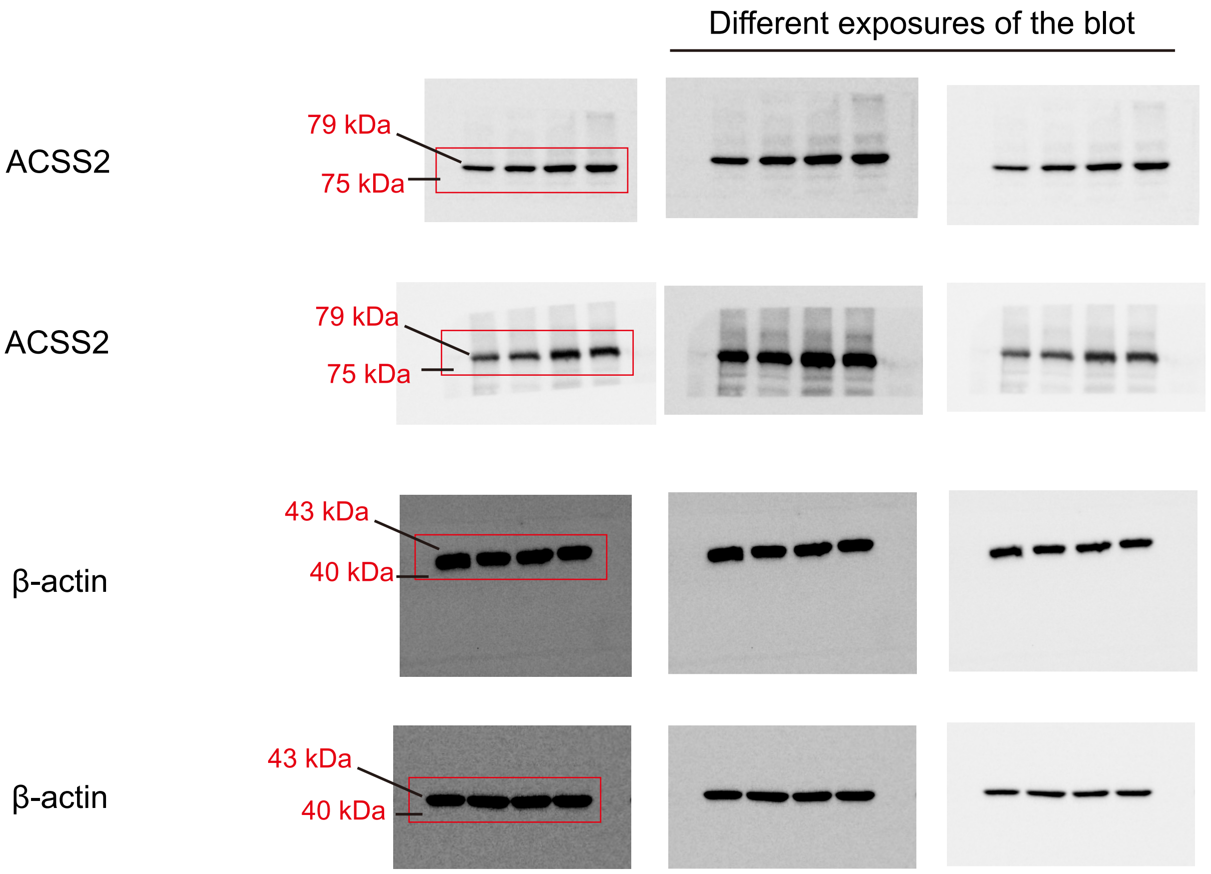

MIAPaCa2

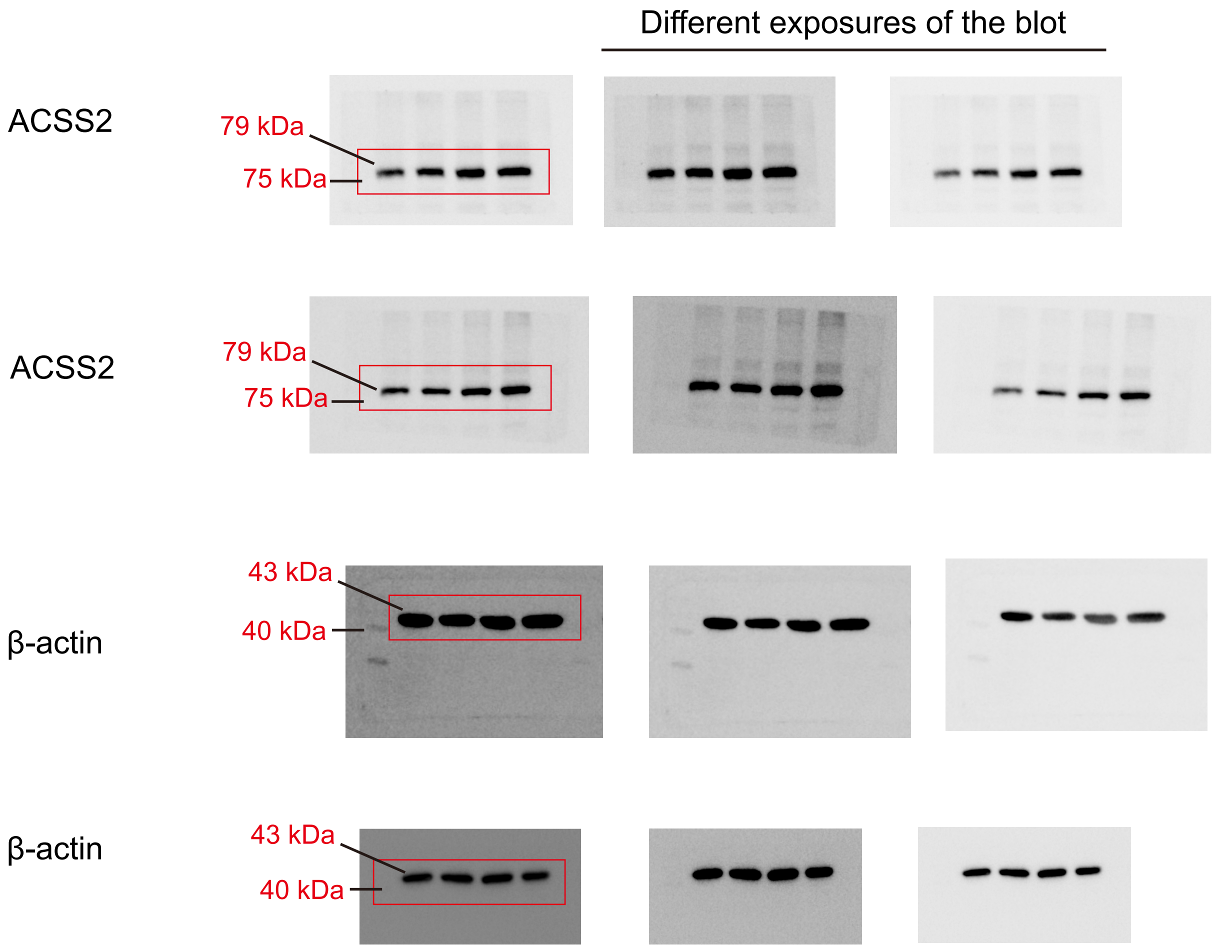

PANC1

Fig 1. Western blot raw data

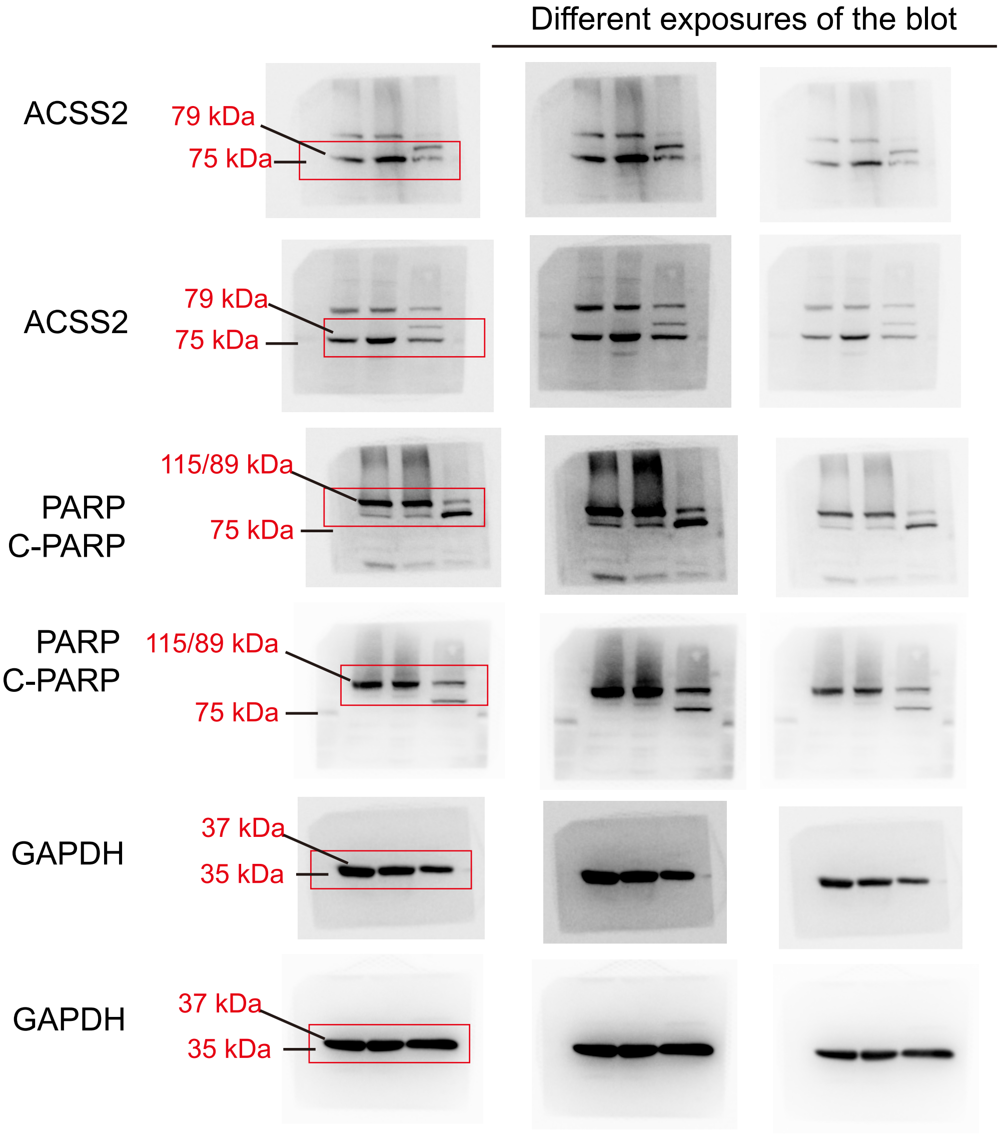

Fig 2. Western blot raw data

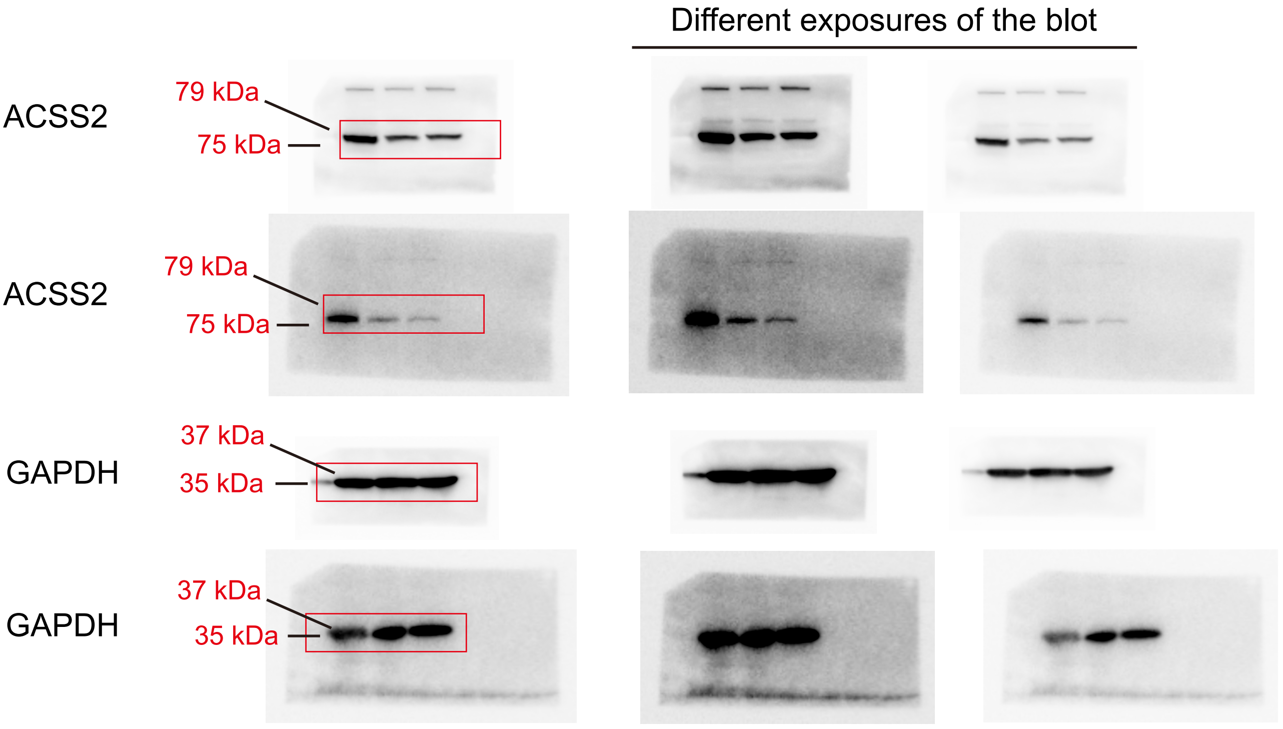

Fig 2. Western blot raw data

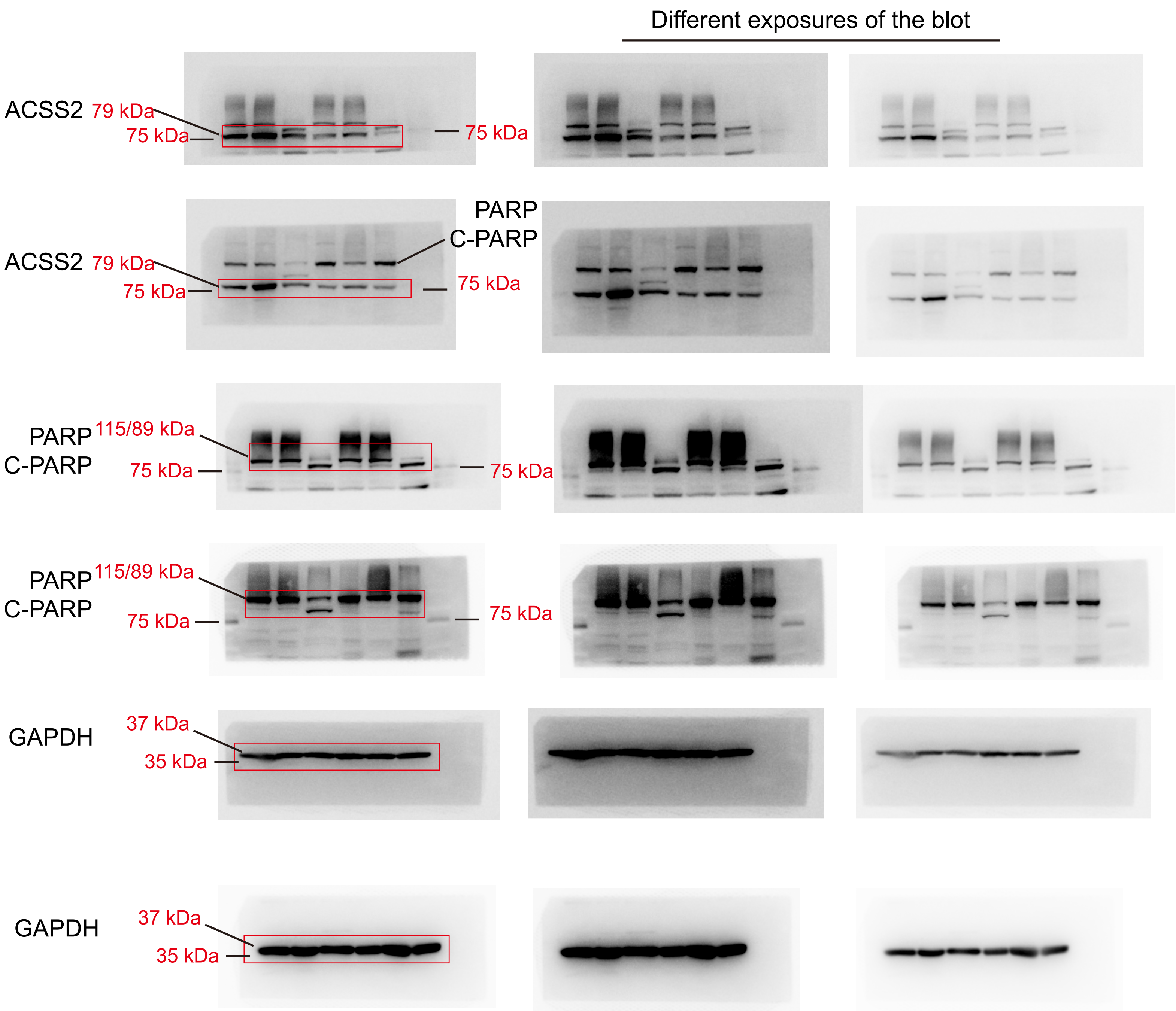

Fig 4. CoIP raw data

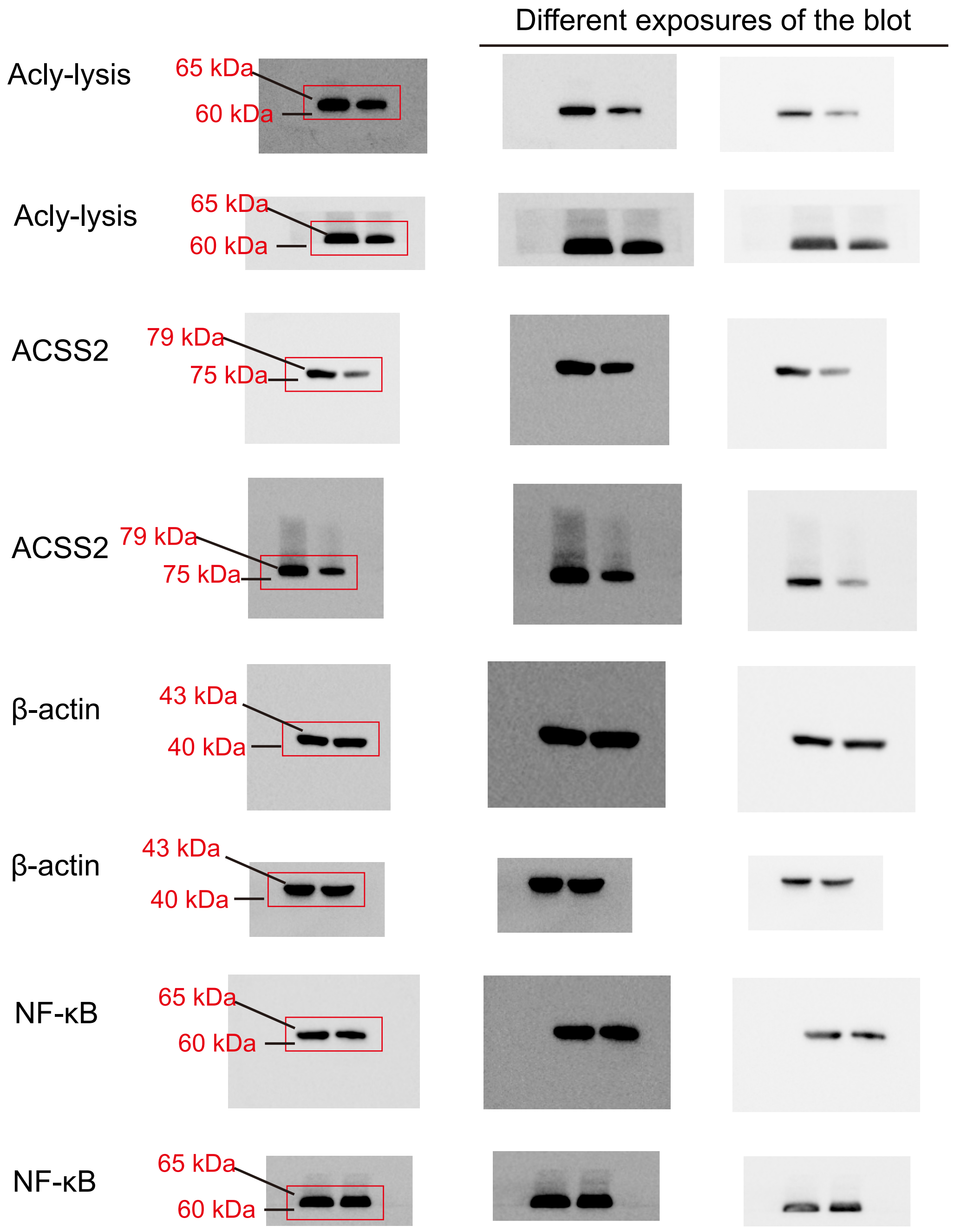

Fig 4. Western blot raw data

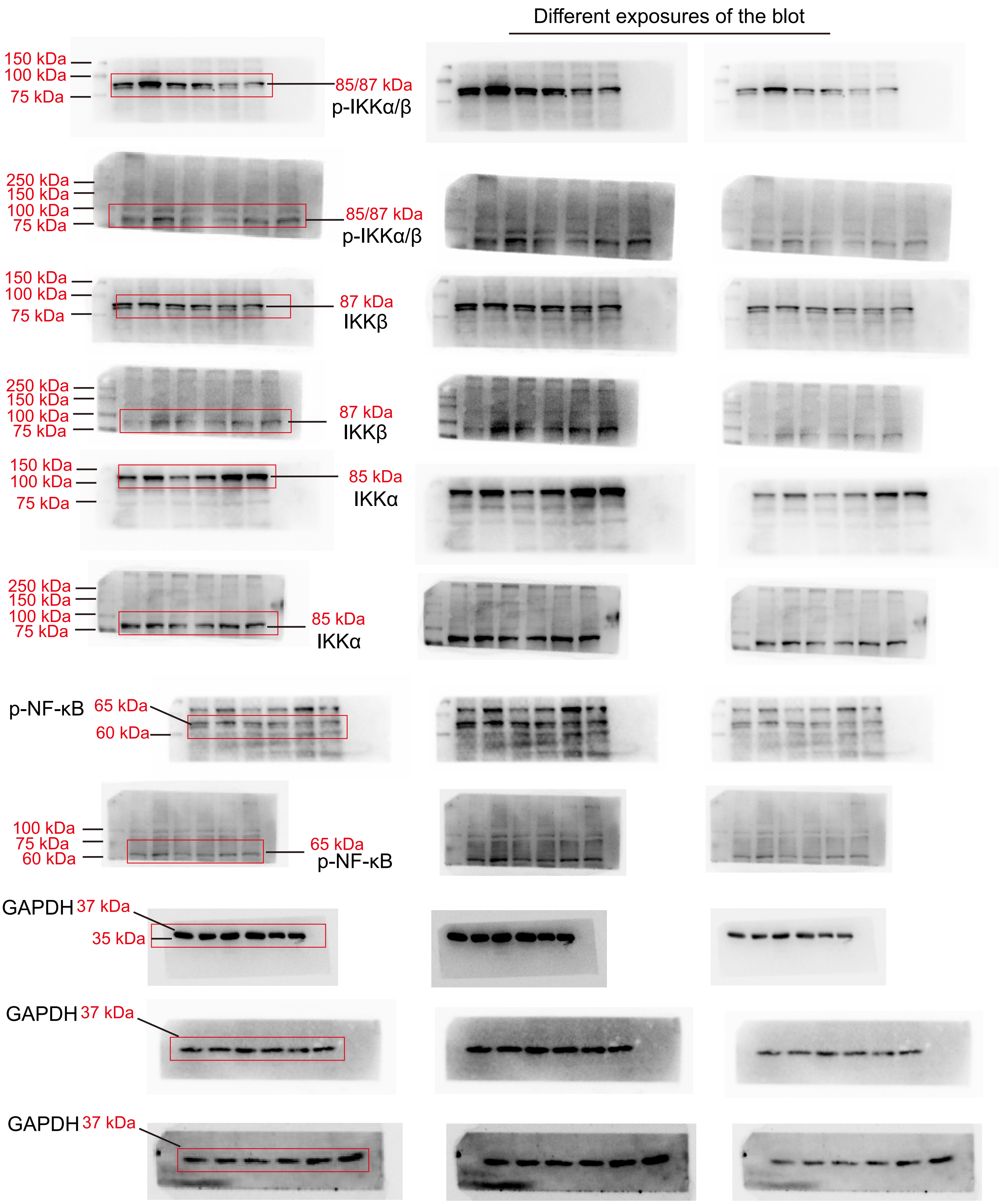

Fig 4. Western blot raw data

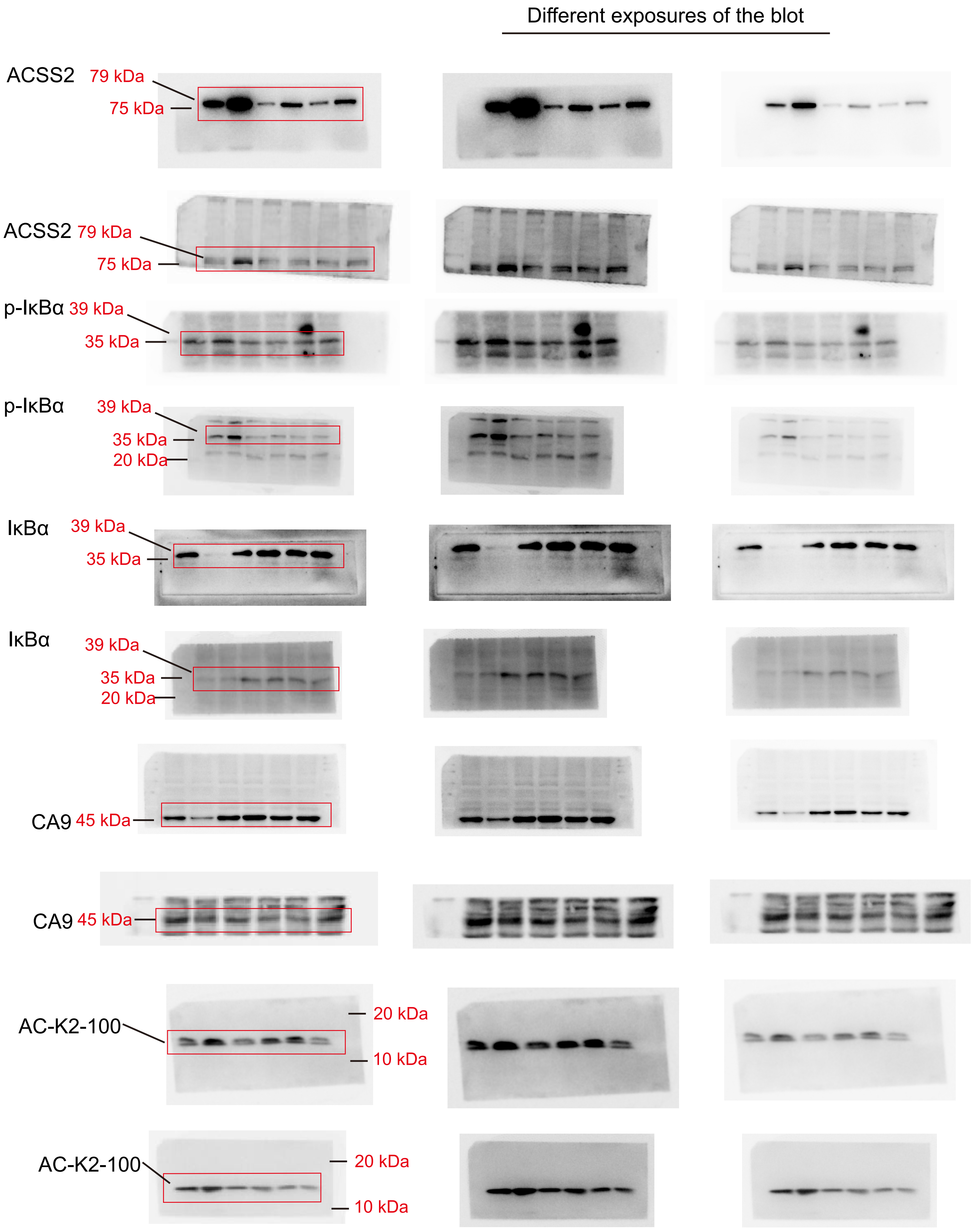

Supplement Fig 3.raw data

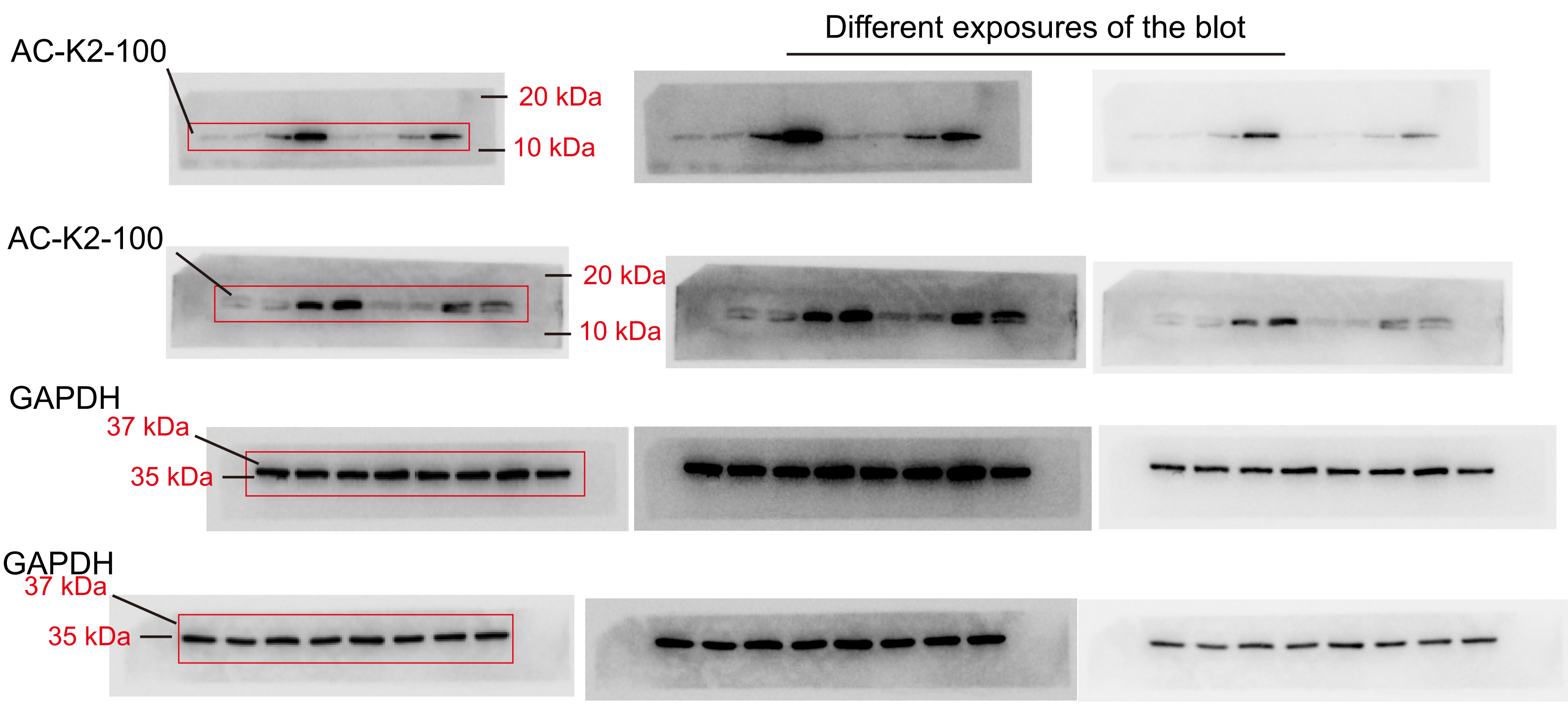

Supplement Fig 4.raw data

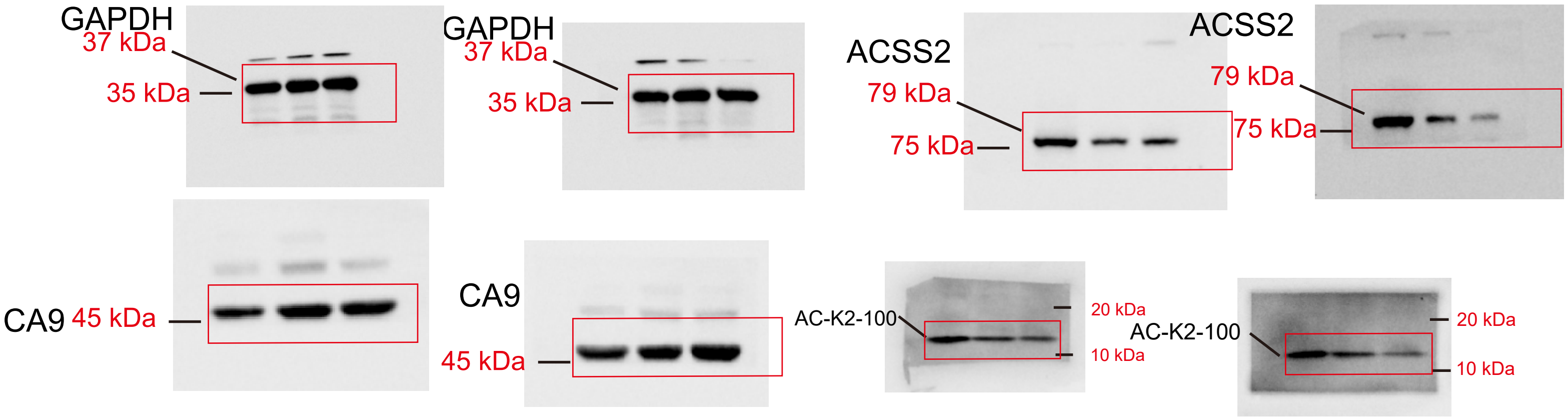

Supplement Fig 5.raw data

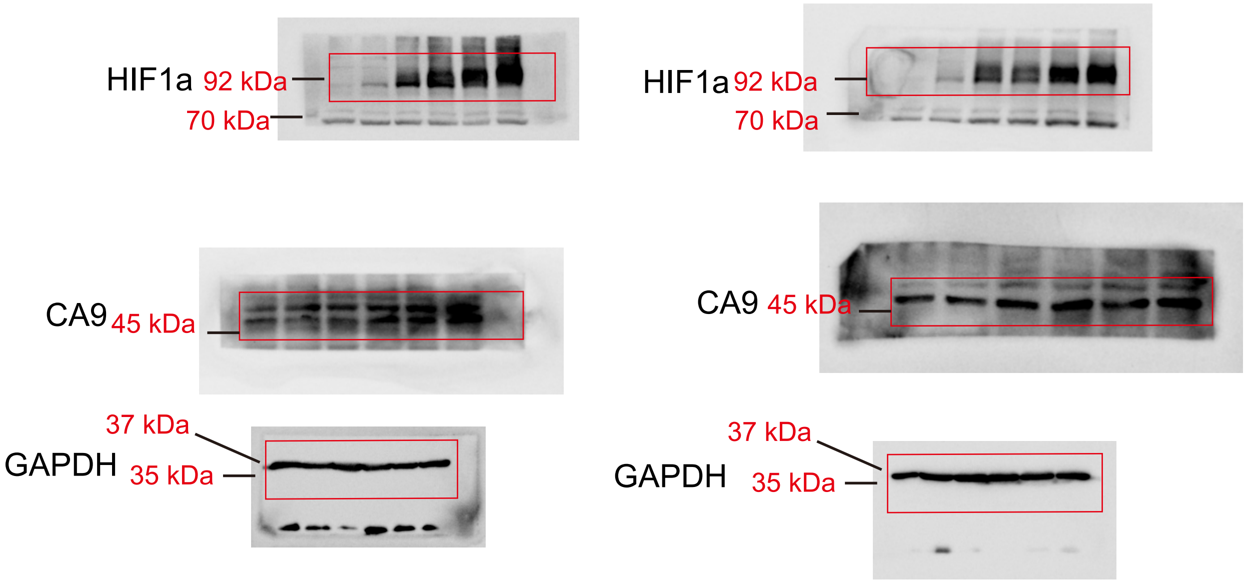

Supplement: Supplementary file 1 — Supplementary Information 1. [file 41598_2023_28261_MOESM1_ESM.pdf]
