# Supplementary material for: ACSS2-mediated NF-κB activation promotes alkaliptosis in human pancreatic cancer cells
Source: Sci Rep. 2023 Jan 27;13:1483. doi: 10.1038/s41598-023-28261-4 (PMC9883393; doi:10.1038/s41598-023-28261-4)
Supplement: Supplementary file 2 — Supplementary Information 2. [file 41598_2023_28261_MOESM2_ESM.pdf]

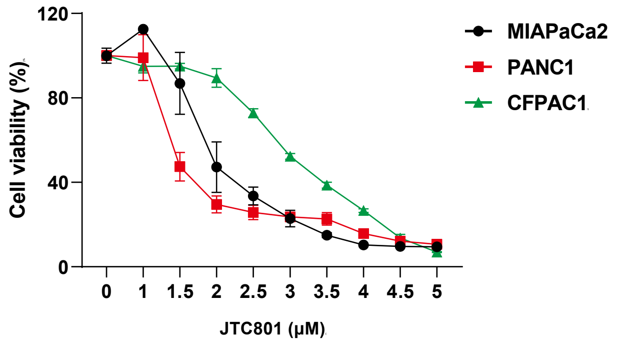

Supplementary Figure 1. Anticancer activity of JTC801 in vitro

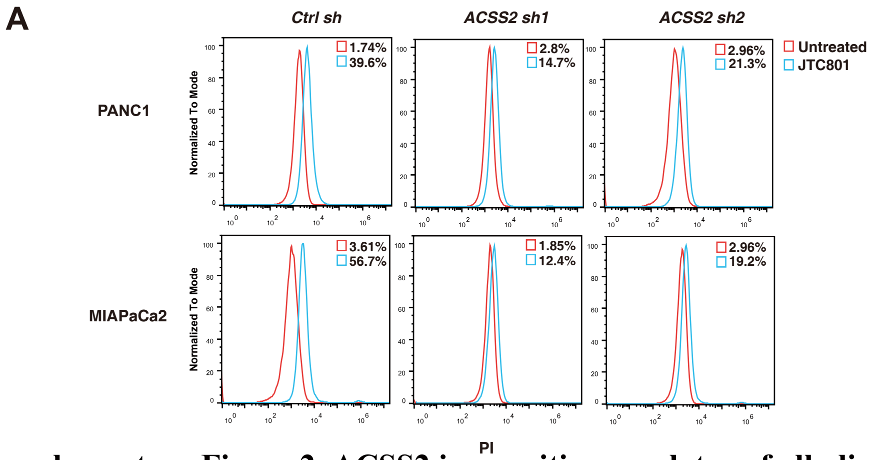

Supplementary Figure 2. ACSS2 is a positive regulator of alkaliptosis.

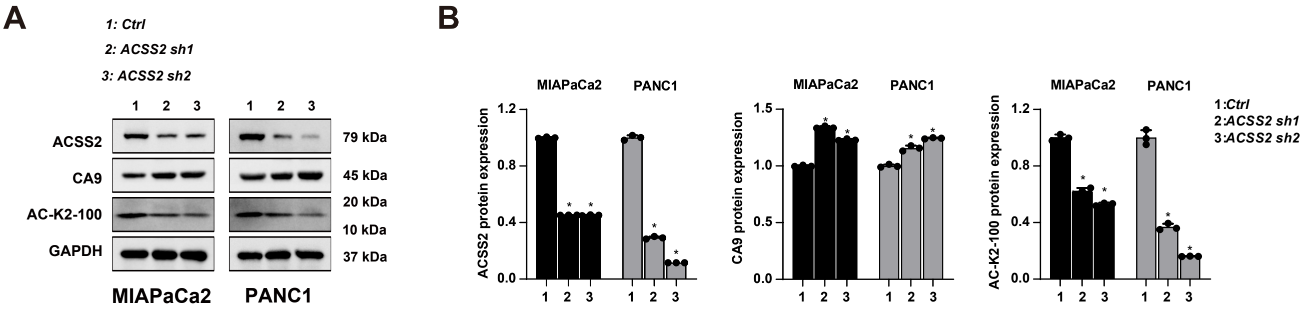

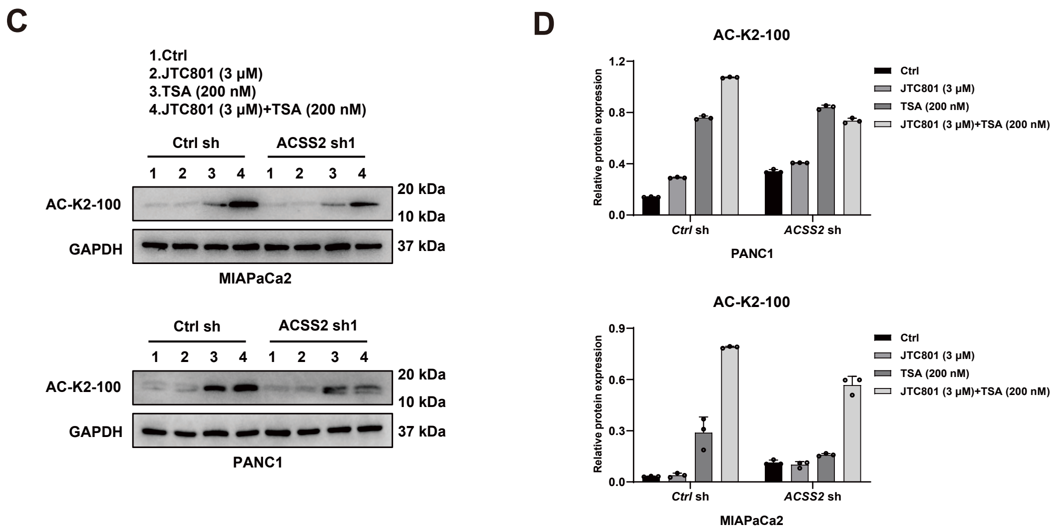

Supplementary Figure 3. Acetylation inhibits CA9 expression and TSA enhances acetylation.

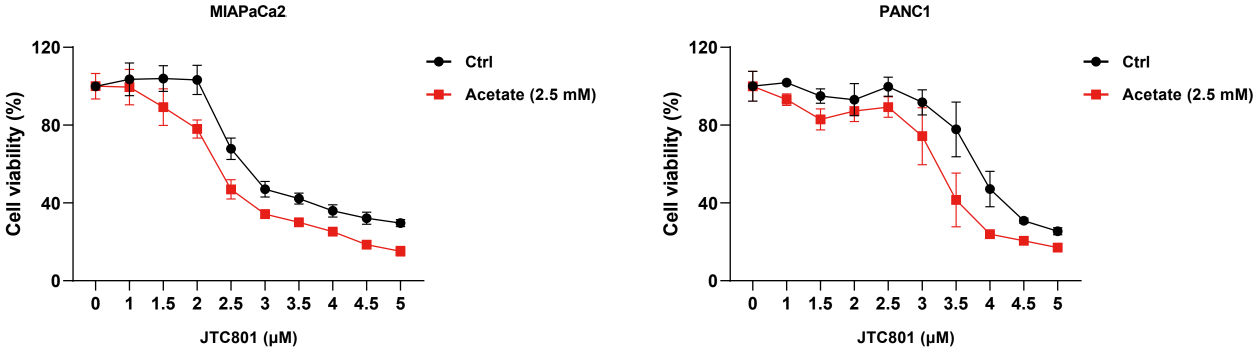

Supplementary Figure 4. Acetate enhances JTC801-induced cell death.

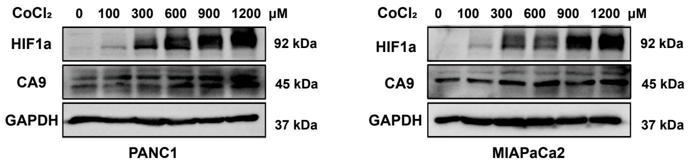

Supplementary Figure 5. CA9 is upregulated during hypoxia
