# Supplementary material for: ACSS2-mediated NF-κB activation promotes alkaliptosis in human pancreatic cancer cells
Source: Sci Rep. 2023 Jan 27;13:1483. doi: 10.1038/s41598-023-28261-4 (PMC9883393; doi:10.1038/s41598-023-28261-4)
Supplement: Supplementary file 3 — Supplementary Information 3. [file 41598_2023_28261_MOESM3_ESM.docx]

**ACSS2-mediated NF-κB activation promotes alkaliptosis in human pancreatic cancer cells**

Dongwen Que^1^, Feimei Kuang^1^, Rui Kang^2^, Daolin Tang^2*^, Jiao Liu^1*^

**Supplementary Information**


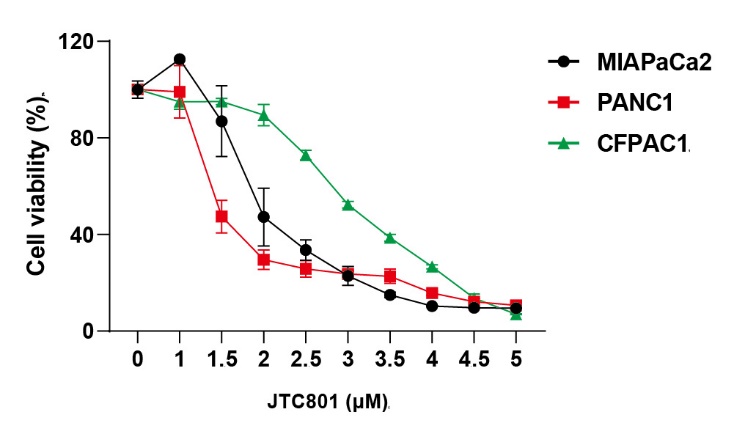


**Supplementary Figure 1. Anticancer activity of JTC801 in vitro.** Indicated human PDAC cell lines were treated with JTC801 (1-5 μM) for 24 hours.


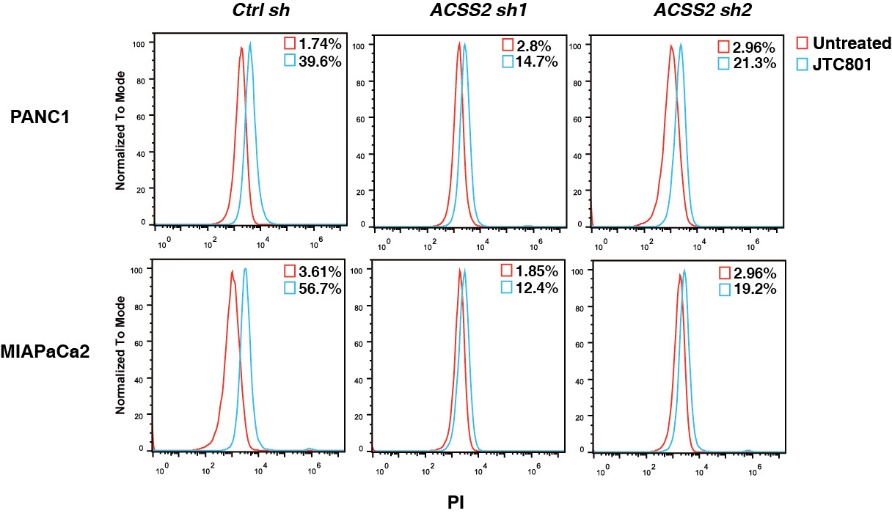


**Supplementary Figure 2. ACSS2 is a positive regulator of alkaliptosis.** Flow cytometeric analysis of cell death by PI staining in indicated cells following treatment with JTC801 (3 µM) for 24 hours.


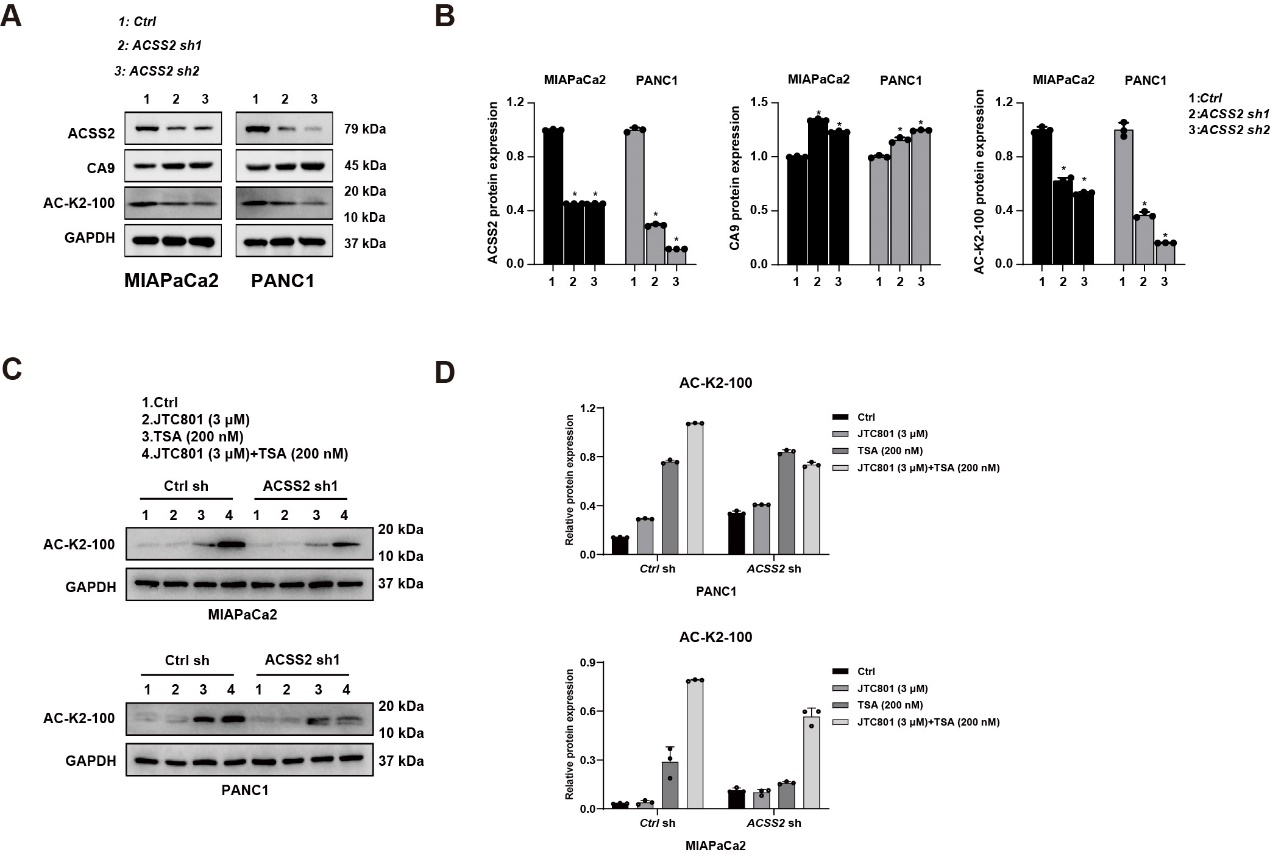


**Supplementary Figure 3. Acetylation inhibits CA9 expression and TSA enhances acetylation.** (A-B) Western blot analysis of ACSS2, CA9 and AC-K2-100 protein expression in indicated *ACSS2* knockdown cell lines (n = 3 biolo­­­gically independent samples). The membrane were cropped and probed for indicated antibodies. Quantitative results are plotted in the right panel. (C-D) Western blot of acetylated extracted from indicated cells following treatment with JTC801 (3 µM) or TSA (200 nM) for 24 hours. Representative western blot probed with indicated antibodies are shown­­ in the top panel. Quantitative results are plotted in the right panel.


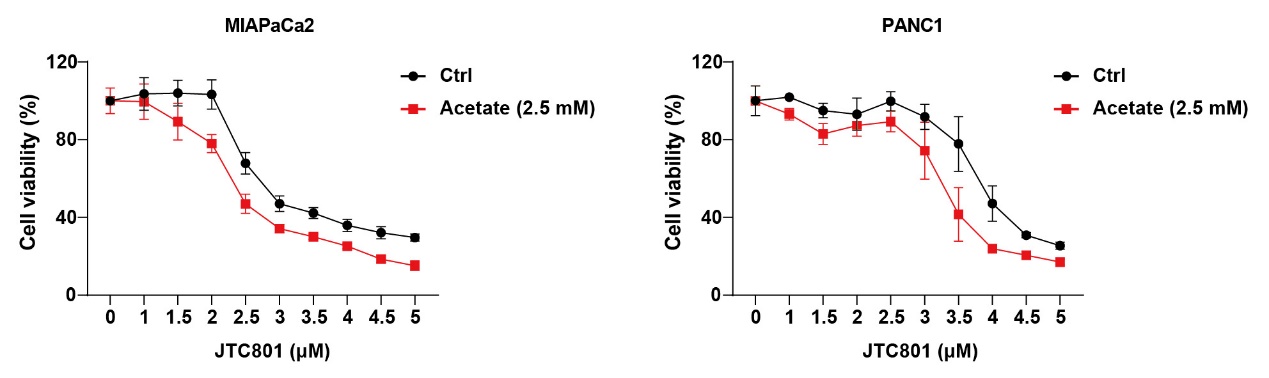


**Supplementary Figure 4.** **A****cetate enhances JTC801-induced cell death.** Indicated PDAC cells treated with JTC801 (1-5 μM) in the absence or presence of acetate (2.5 mM) cell viability were assayed.


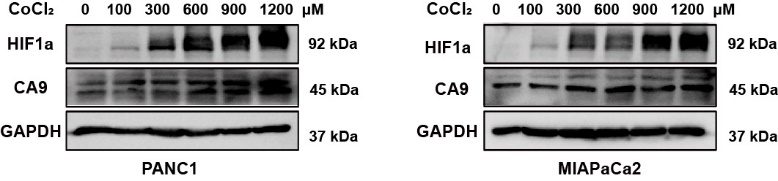


**Supplementary Figure 5. CA9 is upregulated during hypoxia.** Western blot analysis of HIF1a, CA9 and GAPDH protein expression in indicated cell lines treated with CoCl_2_ (0-1200 μM) for 24 hours.
